# Prevalence of and risk factors for hallux rigidus: a cross-sectional study in Japan

**DOI:** 10.1186/s12891-021-04666-y

**Published:** 2021-09-13

**Authors:** Yoshiyuki Senga, Akinobu Nishimura, Naoya Ito, Yukie Kitaura, Akihiro Sudo

**Affiliations:** 1 Departments of Orthopaedic Surgery, Mie University Graduate School of Medicine, 2-174 Edobashi, Tsu city, Mie 514-8507 Japan; 2 Department of Orthopaedic Surgery, Ise Red Cross Hospital, 1-471-2 Funae, Ise city, Mie 516-8512 Japan; 3 Department of Orthopaedic and Sports Medicine, Mie University Graduate School of Medicine, 2-174 Edobashi, Tsu city, Mie 514-8507 Japan

**Keywords:** Hallux rigidus, Cohort study, Epidemiology

## Abstract

**Background:**

Hallux rigidus (HR) is a common osteoarthritis of the first metatarsophalangeal joint. However, the epidemiology and risk factors of this pathology have yet to be clarified.

**Methods:**

We have been conducting cohort studies among individuals over 50 years old every 2 years since 1997. This study analyzed data from the 7th to 10th checkups in 2009, 2011, 2013, and 2015. We investigated the prevalence of HR and its risk factors in a total of 604 individuals (mean age, 67.1 ± 6.4 years; 208 men, 396 women). Radiographic HR was defined as Hattrup and Johnson classification grade 1 or higher. Knee osteoarthritis (KOA) was scored according to the Kellgren-Lawrence grading system. Radiographic KOA was defined as grade 2 or higher. Cases with a hallux valgus (HV) angle of 20° or higher were defined as showing HV. Statistical analyses were performed using the Kruskal-Wallis test, Fisher’s exact test, logistic regression modeling, and the Cochran-Armitage trend test. All *p-*values presented are two-sided and values of *p* < .05 were considered statistically significant.

**Results:**

The prevalence of HR was 26.7% (161/604). Rates of grade 0, 1, 2, and 3 HR according to the Hattrup and Johnson classification were 73.3% (443/604), 16.4% (99/604), 8.0% (48/604), and 2.3% (14/604), respectively. Overall ratio of symptomatic HR was 8.1%. Univariate analysis revealed KOA, gout attack (GA), and HV as significantly associated with HR. The same factors were confirmed as independent risk factors for HR in multivariate analysis. All parameters were significantly associated with HR. Odds ratios of KOA, HV, and GA for HR were 1.73, 3.98, and 3.86, respectively. The presence or absence of KOA was significantly associated with severity of HR.

**Conclusions:**

This study revealed that the prevalence of HR in the elderly (≥50 years) was 26.7%. KOA, HV, and GA were independent risk factors for HR. KOA was associated with severity of HR.

## Background

Hallux rigidus (HR) is an osteoarthritis of the first metatarsophalangeal (MTP) joint, and the most common arthritic condition affecting the foot [1]. HR causes various symptoms, including local pain in the 1st MTP joint, plantar calluses, stiffness, and enlargement of the joint [2]. Radiographically, HR is characterized by joint space narrowing, osteophytic lipping of the metatarsal head and proximal phalanx, and sesamoid hypertrophy [3]. While arthritis can be caused by traumatic or iatrogenic injuries that directly damage the articular cartilage of the MTP joint, the most common etiology of HR is idiopathic [1].

HR was initially described by Davies-Colley in 1887 [4]. While the pathology is sometimes seen in daily medical practice, its prevalence and epidemiology remain unclear. Previous studies have reported a high prevalence of HR (20–35%) [5–8], and an extremely high prevalence (61%) among the population aged over 80 years [7]. However, those studies just examined the prevalence of HR among patients with systematic osteoarthritis. Furthermore, few well-designed studies have investigated risk factors for HR, although numerous risk factors have been proposed [2]. An in-depth study of HR as diagnosed using the Hattrup and Johnson classification was thus required to clarify the prevalence and risk factors of this entity.

The purpose of this cross-sectional study of a population sample in Japan was to investigate the prevalence of HR and its risk factors among Japanese community dwellers.

## Methods

### Sample collections

We have been conducting a cross-sectional study among individuals over 50 years old to investigate factors associated with orthopedic-related diseases such as knee osteoarthritis (KOA) and osteoporosis every 2 years since 1997 [9, 10]. The present study analyzed individuals recruited from residents of a mountain village in Japan. All investigations were conducted at the local hospital. Before presenting for direct examination, a baseline questionnaire was sent to each participant. Participants answered several questions on age, sex, history of smoking and drinking, foot pain, and medical history, including hypertension, hyperlipidemia, diabetes mellitus (DM), and gout attack (GA). We defined individuals over 65 years old as elderly. In the Research on Osteoarthritis Against Disability study, knee pain was defined as pain occurring in and around the knee joint on most days during the past month, and the same definition was applied in the present study to define foot pain that has continued longer than 1 month [11]. Symptomatic HR was defined as radiographic HR in a patient reporting foot pain. Direct examination consisted of physical measurements of height and weight, a medical interview, a physical examination by an orthopedic surgeon, and X-rays. In the case of X-ray images that could not be evaluated, images were taken again on the spot. Body mass index (BMI) was calculated. Participants also underwent bone mineral densitometry of the forearm to screen for osteoporosis, defined as a bone mineral density T-score of − 2.5 or below [12].

### Participants

**Fig. 1:**
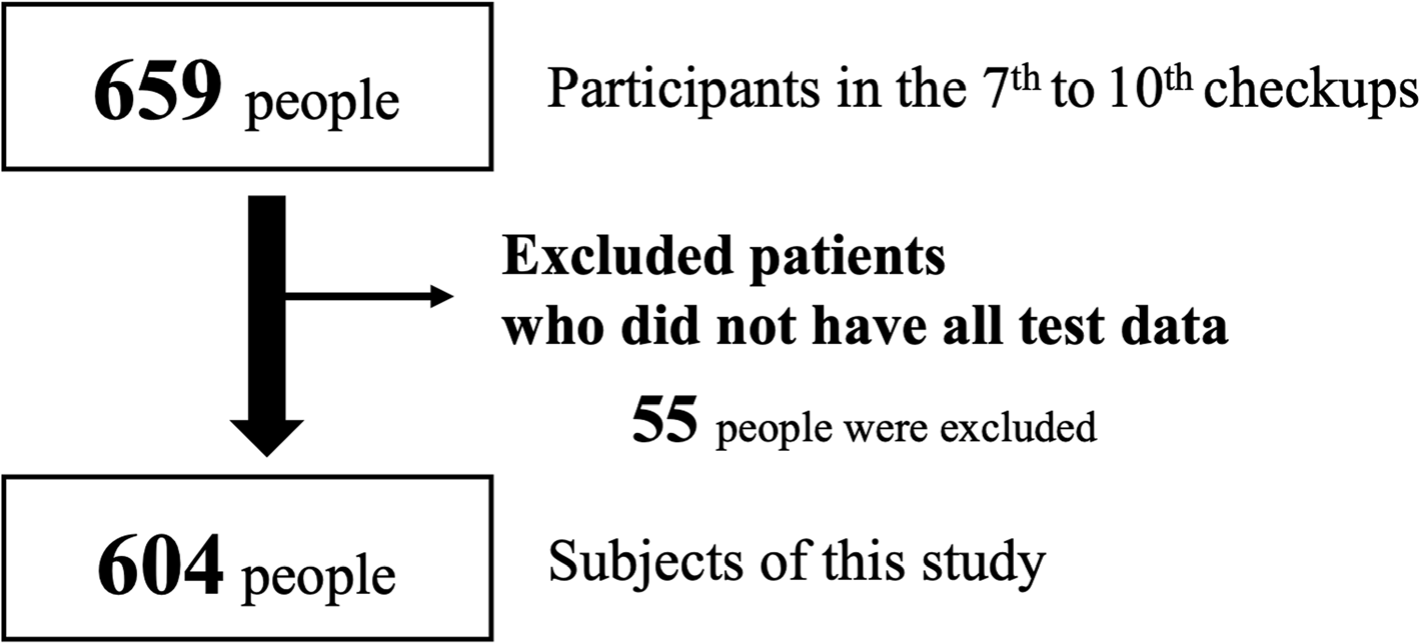
Flowchart of the selection process for the subjects in this study

This study analyzed data from the 7th to 10th checkups, conducted in 2009, 2011, 2013, and 2015, respectively. For those who participated in more than one of these four checkups, only data from the first checkup were included. However, those for whom all screening data were unavailable were excluded. As shown in Fig. 1, 55 of the initial 659 individuals were excluded, leaving a final total of 604 individuals to participate in the study (mean age, 67.1 ± 6.4 years; 208 men, 396 women). We divided participants into HR (+) and HR (−) groups according to the presence or absence of HR.

All procedures performed with participants were conducted in accordance with the ethical standards of the institutional and/or national research committee, and with the Declaration of Helsinki (1964) and its later amendments or comparable ethical standards. Written informed consent was obtained from all participants before enrollment.

### Definitions of KOA and HR

**Fig. 2:**
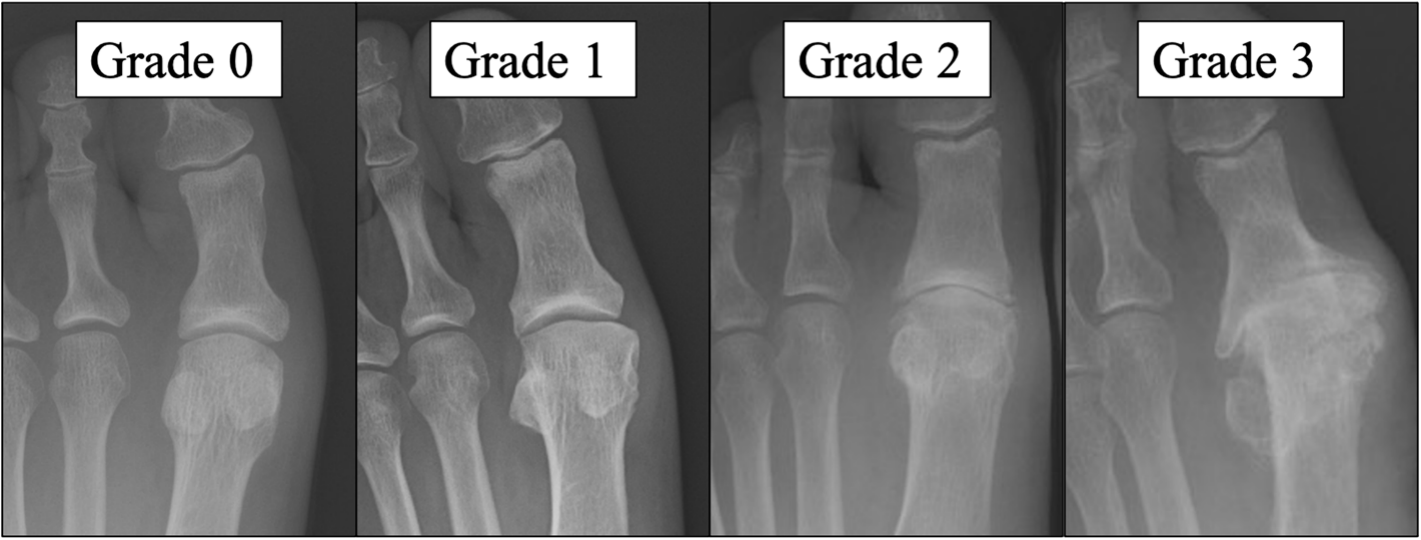
Severity of hallux rigidus. Grade 0: Normal. Grade 1: Preservation of joint space, mild osteophyte formation. Grade 2: Mild to moderate joint-space narrowing, moderate osteophyte formation, subchondral sclerosis and cysts. Grade 3: Severe joint-space narrowing, significant osteophyte formation, loose bodies, subchondral sclerosis and cysts

Knee X-rays were taken with the patient standing, knee fully extended. KOA was scored according to the Kellgren-Lawrence grading system [13]. Radiographic KOA was defined as grade 2 or higher. We also took foot X-rays with the participant standing upright with both feet on the cassette, as described by Saltzman et al. [14]. Severity of HR was scored based on the modified version of the Hattrup and Johnson classification [15]. Accordingly, severity of HR was classified as: Grade 0, normal; Grade 1, preservation of joint space, mild osteophyte formation; Grade 2, mild to moderate joint space narrowing, moderate osteophyte formation, subchondral sclerosis and cysts; and Grade 3, severe joint space narrowing, significant osteophyte formation, loose bodies, subchondral sclerosis and cysts (Fig. 2). Radiographic HR was defined as grade 1 or higher, with normal appearance classed as grade 0. Cases with a HV angle of 20° or higher were defined as showing HV.

### Statistical analysis

The primary objective of this study was to clarify the prevalence of HR among individuals who were more than 50 years old. The secondary objective was to explore risk factors for HR. We compared characteristics using the Kruskal-Wallis test for continuous variables and Fisher’s exact test for categorical variables. Logistic regression modeling was used to examine the relationship between variables and HR. First, a univariate analysis was performed. Multivariate analysis was then performed by entering those factors showing significant differences in the univariate analysis and adjusting for age and sex. The Cochran-Armitage trend test was used to examine the relationships between risk factors and severity of HR in a linear trend. All statistical analyses were performed using R version 3.3.2 statistical software (R Foundation for Statistical Computing, Vienna, Austria). All *p-*values presented are two-sided and values of *p* < .05 were considered statistically significant.

## Results

### Prevalence of HR and participant characteristics

**Fig. 3:**
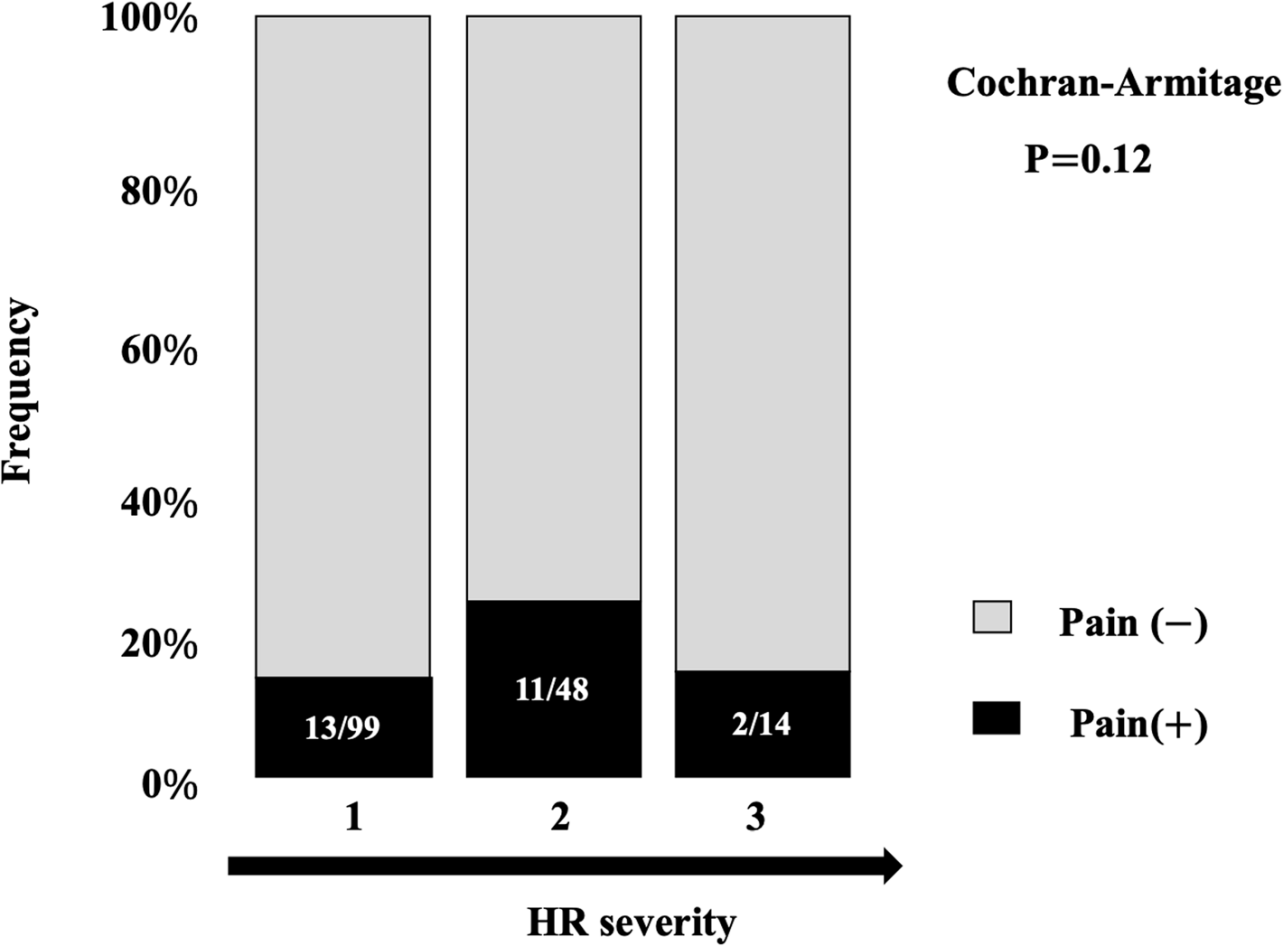
Ratio of symptomatic hallux rigidus according to severity

The prevalence of HR as diagnosed by X-ray examination was 26.7% (161/604) among the entire cohort. Rates of HR grades 0, 1, 2, and 3 were 73.3% (443/604), 16.4% (99/604), 8.0% (48/604), and 2.3% (14/604), respectively. The overall ratio of symptomatic HR was 16.1% (26/161) and the percentage of symptoms did not appear associated with severity of HR (Fig. 3). Table 1 shows a comparison of participant characteristics between groups. No significant differences in various factors were seen between groups, except for KOA, HV, diabetes mellitus, and GA. Specifically, the results showed that patients with KOA, HV, DM, and GA were more likely to have HR.

**Table 1:** Participant characteristics according to the presence or absence of hallux rigidus

Variable	Hallux rigidus (-) *n*=443	Hallux rigidus (+) *n*=161	*p*-value
Age (mean years ± SD)	66.9± 6.7	67.6 ± 5.5	0.32
Age (≥65)	326 (73.6%)	130 (80.7%)	0.087
Sex (female)	286 (64.6%)	110 (68.3%)	0.44
BMI	23.4 ± 3.3	23.7 ± 3.1	0.49
BMI (≥25)	117 (26.4%)	56 (34.8%)	0.053
Knee osteoarthritis	93(21.6%)	68 (39.1%)	*<.001
Hallux valgus	100(22.6%)	87 (54.0%)	*<0.01
Heberden's node	147 (33.2%)	64 (39.8%)	0.24
Vertebral fracture	62 (14.0%)	32 (19.9%)	0.12
Osteoporosis	106 (23.9%)	45 (28.0%)	0.29
Hypertension	172 (38.8%)	71 (44.1%)	0.26
Diabetes mellitus	33 (7.4%)	21 (13.0%)	*0.037
Hyperlipidemia	171 (38.6%)	57 (35.4%)	0.51
Gout attack	8 (1.8%)	8 (5.0%)	*0.044
Smoking	78 (17.6%)	29 (18.0%)	0.90
Alcohol	152 (34.3%)	52 (32.3%)	0.70

*Abbreviations SD* standard deviation, *BMI* body mass index

*p < .05

### Risk factors for HRs

We next examined risk factors for HR. Table 2 shows the results of uni- and multivariate analyses for predictors of HR among the population. Univariate analysis revealed BMI, KOA, HV, diabetes mellitus, and GA as significantly associated with HR. Multivariate analysis confirmed KOA, HV, and GA as independent risk factors for HR (KOA: odds ratio (OR) 1.73, 95% confidence interval (CI) 0.80–1.85, *p* < .05; HV: OR 3.98, 95%CI 2.68–5.92, *p* < .05; GA: OR 3.86, 95%CI 1.24–12.0, *p* < .05). KOA, HV, and GA thus appear to be independent HR-related factors, suggesting that they may be independent risk factors for HR.

**Table 2:** Uni- and multivariate analyses of risk factors for hallux rigidus, adjusted for age and sex

	Univariate		Multivariate	
Variable	OR (95% CI)	*p*-value	OR (95% CI)	*p*-value
Age (≧65)	1.51 (0.96–2.35)	0.072		
Sex (female)	1.18 (0.81–1.74)	0.39		
BMI (≧25)	1.49 (1.01–2.19)	*0.045	1.10 (0.72–1.70)	0.66
Knee osteoarthritis	2.32 (1.59–3.40)	* < .001	2.08 (1.36–3.17)	* < .001
Hallux valgus	4.03 (2.75-5.91)	* < .001	3.96 (2.63-5.96)	* < .001
Heberden's node	1.30 (0.86–1.97)	0.22		
Vertebral fracture	1.49 (0.91–2.44)	0.12		
Osteoporosis	1.26 (0.84–1.89)	0.27		
Hypertension	1.24 (0.86–1.79)	0.25		
Diabetes mellitus	1.86 (1.04–3.33)	*0.036	1.87 (0.99–3.52)	0.053
Hyperlipidemia	0.87 (0.59–1.26)	0.45		
Gout attack	2.83 (1.04–7.67)	*0.041	3.16 (1.05–9.52)	*0.041
Smoking	1.03 (0.64–1.65)	0.91		
Alcohol	0.91 (0.62–1.34)	0.64		

*Abbreviations*: *OR* odds ratio, *CI* confidence interval, *BMI* body mass index

*p < .05

### Relationship between KOA and severity of HR

**Fig. 4:**
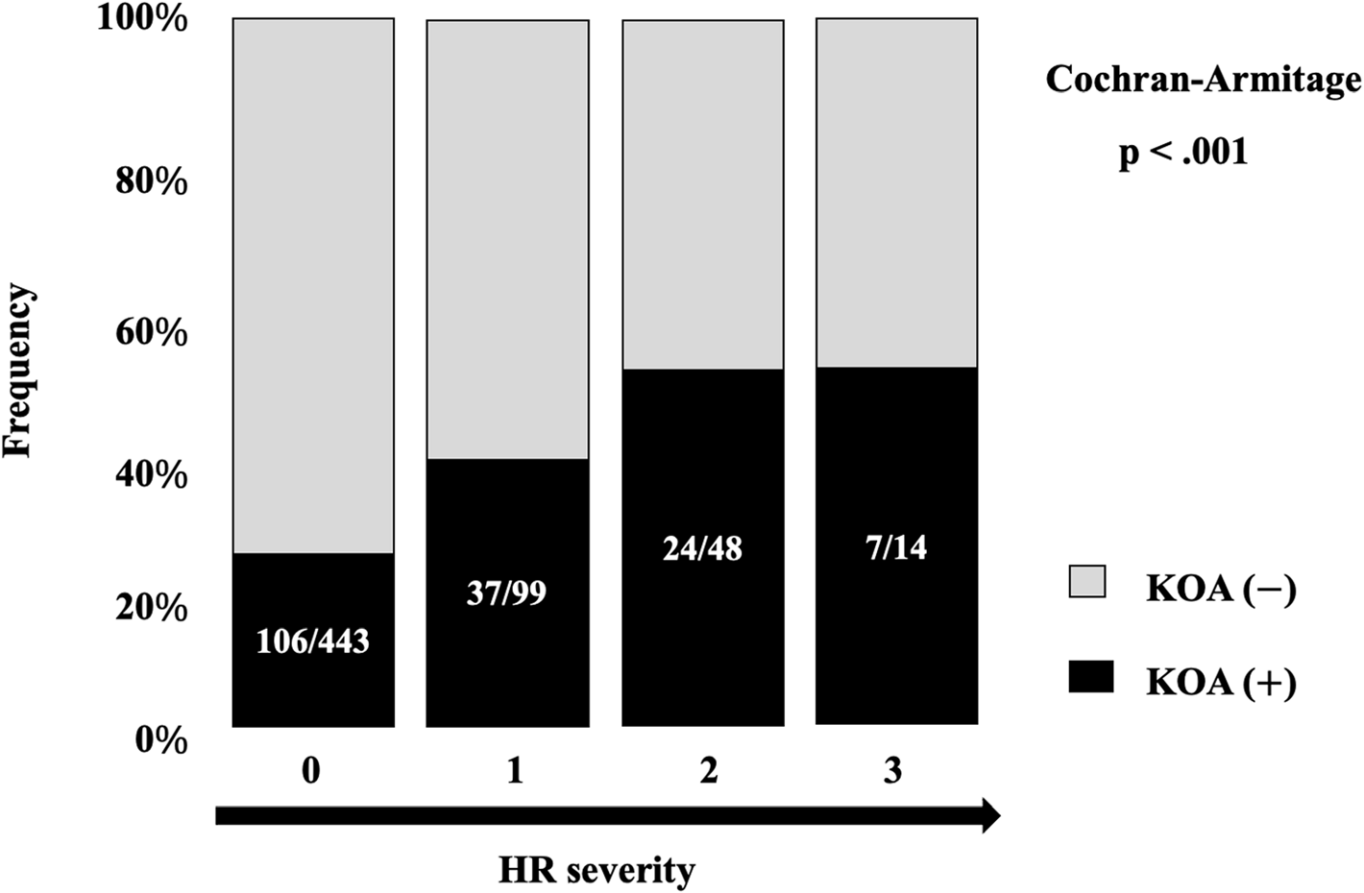
Relationship between hallux rigidus severity and knee osteoarthritis

We subsequently examined whether these risk factors were associated with severity of HR. While HV and GA were not associated with severity of HR, KOA was associated with the severity of HR. Figure 4 shows the relationship between KOA and the severity of HR. KOA is significantly associated with the severity of HR. The frequencies of grades 0, 1, 2, and 3 were 23.9% (106/443), 37.4% (37/99), 50.0% (24/48), and 50.0% (7/14), respectively. The frequency of KOA was significantly associated with the severity of HR (*p* < .001). Patients with KOA may thus be more likely to have severe HR.

## Discussion

We investigated the prevalence of HR and its risk factors among individuals over 50 years old living in a village in Japan. We identified three important clinical issues: 1) the prevalence of HR among this sample of a general population over 50 years old was 26.7%; 2) KOA, HV, and GA were independent risk factors for HR; and 3) KOA was associated with severity of HR.

The 26.7% prevalence of HR is similar to findings from previous studies (20–35%) [5–8]. In most such studies, HR was diagnosed using the Kellgren-Lawrence grading system, whereas the present investigation diagnosed HR using the modified Hattrup and Johnson classification. Grade 2 in the Kellgren-Lawrence grading system is almost identical to Grade 1 in the modified Hattrup and Johnson classification. The cut-off criterion for diagnosing HR in this study thus resembled those in previous reports. This is presumably one reason the prevalence of HR in this study was similar to those in previous reports.

Several risk factors have been proposed in the literature, including female sex [16], history of trauma [17], rheumatoid arthritis [18], long proximal phalanx of the hallux [19], varus deformities of the forefoot or rearfoot [19], HV deformity [20], soft-tissue contracture [21], short or long first metatarsal [22], increased interphalangeal angle of the hallux [23], family history [16], and ill-fitting footwear [24]. However, none of these risk factors were determined from epidemiological studies of local residents. HV, KOA, and GA were identified as independent risk factors in this study. Previous reports have clarified that KOA is related to HV [25]. Interestingly, our study demonstrated KOA as an independent risk factor for HR, regardless of HV. Further, the severity of HR was higher in patients with KOA. This may reflect individuals with a genetic predisposition to cartilage damage developing KOA and HR. This possibility is strengthened by a study revealing that OA of the 1st MTP was associated with OA at differing sites, including the knee and interphalangeal joints [8]. In short, we confirmed a close relationship between KOA and HR in the present study. Increased BMI is known to be associated with radiological findings and symptoms of KOA. Similarly in this study, BMI was significantly associated with KOA. However, BMI was not associated with the severity of HR [26].

This study revealed HV as an independent risk factor for HR. HV and HR are two common diseases that affect the 1st MTP joint. Despite affecting the same joint, the clinical and pathological profiles of HV and HR are quite different, and patients are generally accepted to develop either primary HR or HV, not both [27, 28]. However, end-stage HV may be associated with the development of arthritis and may lead to HR.

Mertz reported that the individuals with HR experienced GA more often than those without HR [29]. GA has also been reported to cause inflammation of the 1st MTP joint and erosion of the bone, which may result in HR [30]. Those hypotheses were supported by our study clarifying a history of GA as a strong risk factor for HR.

Several limitations to this study should be considered when interpreting the results. First, this study involved medical checkups in the limited local area of a mountainous, rural area in Japan, and the participants are not representative of the entirety of Japan. Second, this study was cross-sectional in design, and the results thus cannot be used to determine whether risk factors such as KOA cause HR or vice versa. Third, participants were relatively healthy elderly individuals, because they were able to walk to the local hospital. Fourth, we only obtained anteroposterior X-rays of the feet with the participant standing upright; lateral-view images were not taken. Fifth, increased pronation of the hindfoot leads to instability of the first joint, predisposing the joint to degenerative processes, although the presence of flat feet was not considered in this investigation.

## Conclusions

We retrospectively examined the prevalence of HR and sought to identify associated risk factors among individuals over 50 years old. We made three important clinical findings: the prevalence of HR among individuals over 50 years old was 26.7%; KOA, HV, and GA were independent risk factors for HR; and KOA was associated with the severity of HR. Larger, longitudinal studies are needed to confirm our findings.

## Data Availability

The datasets analyzed during the current study are available from the corresponding author on reasonable request.

## Abbreviations

HR: Hallux rigidus
MTP: Metatarsophalangeal
KOA: Knee osteoarthritis
DM: Diabetes mellitus
GA: Gout attack
HV: Hallux valgus
BMI: Body mass index
OR: Odds ratio
CI: Confidence interval
